# Identification and Functional Characterization of the *CrRLK1L* Gene Family in Salt Tolerance in Rice (*Oryza sativa* L.)

**DOI:** 10.3390/genes16121454

**Published:** 2025-12-04

**Authors:** Haoqiang Du, Xingyu Wang, Jifang Hu, Kefei Tan, Chuanzeng Liu, Bo Ma

**Affiliations:** 1 Qiqihar Branch of Heilongjiang Academy of Agricultural Sciences, Qiqihar 161006, China; 2 Northeast Branch of National Saline–Alkali-Tolerant Rice Technology Innovation Center, Harbin 150000, China

**Keywords:** rice (*Oryza sativa* L.), *CrRLK1L* gene family, expression pattern, salt stress

## Abstract

**Background:** As key members of the plant receptor-like kinase family, rice *CrRLK1Ls* play diverse roles in plant growth, development, and stress responses. Although rice *CrRLK1Ls* have been initially characterized, our understanding of their functions remains limited. **Methods:** We identified *OsCrRLK1L* genes via Hidden Markov Model (HMM) searches against the rice genome. Subsequent analyses encompassed their physicochemical properties, chromosomal distribution, gene structure, phylogenetic relationships, conserved domains, and cis-acting elements.Salt-responsive candidates were screened using a GEO dataset, and their expression profiles were validated under salt stress using quantitative real-time PCR. **Result:** A total of 36 *OsCrRLK1L* genes, all containing both Malectin and tyrosine kinase domains, were identified in the rice genome and showed an uneven chromosomal distribution. Phylogenetic analysis classified them into three subclades, with Group II and Group III being specific to rice and *Arabidopsis thaliana*, respectively. Promoter analysis revealed that the promoter regions of these genes contained an abundance of cis-acting elements related to hormone and stress responses. RNA-Seq and enrichment analysis indicated that *OsCrRLK1L* genes exhibit tissue specificity and participate in salt stress responses. Furthermore, *CrRLK1L2* and *CrRLK1L10* showed tissue-specific differential expression under salt stress. **Conclusions:** In summary, our study lays the groundwork for future research into the biological roles of *OsCrRLK1L* genes during salt stress.

## 1. Introduction

A range of stresses in natural environments adversely affect plant growth and development. Thus, the accurate perception of external signals and subsequent responses are critical for their survival [1]. The plant *RLK* gene family is extensive, as evidenced by its large membership of over 600 in *Arabidopsis* and more than 1131 in rice [2,3]. *RLKs* serve as a vast and intricate signaling network that orchestrates multiple physiological processes in plants [4]. As a key *RLK* subgroup, the *CrRLK1L* subfamily regulates plant growth and development, as well as crucial biotic and abiotic stress responses [5].

A defining characteristic of the *CrRLK1L* family is that it contains both Malectin and Tyrosine Pkinase domains [6]. This unique combination of domains confers specific biological functions to the *CrRLK1L* family, consequently enabling it to regulate multiple biological processes [7]. The widespread distribution of *CrRLK1L* members across plant species has been established, with recent studies underscoring their critical contributions to growth, development, immunity, and fertilization [8]. Research has demonstrated that the *CrRLK1L* family is critically involved in stress responses in various crops. In wheat, the 15 characterized *CrRLK1L* members have been implicated in abiotic stress responses, such as those induced by cold, heat, drought, and salt [9]. Overexpression of *GmCrRLK1L2* in soybean impairs salt tolerance [10]. Meanwhile, *CrRLK1L* family members in eggplant display distinct, tissue-specific expression patterns and functions throughout development [11]. Eighty-nine *CrRLK1L* family members in peanuts were identified as being responsive to drought and aluminum stress, with their expression specifically induced by these treatments [12]. Multiple *CrRLK1L* genes in rice function in disease resistance and abiotic stress responses. For instance, increased susceptibility to rice blast fungus is observed in *FLR1* and *FLR13* mutants, whereas *FLR2* and *FLR11* mutants exhibit enhanced resistance [13]. *OsMRLK63* is a typical homolog of *CrRLK1L* that rapidly activates the receptors *OsRALF45* and *OsRALF46*, thereby playing a role in drought tolerance [14]. As the most extensively studied *CrRLK1L* protein in *Arabidopsis*, *AtFER* was originally identified in pollen tube mutants. Functioning as a receptor for signaling peptides such as *RALF1*, and in addition to its roles in various hormonal pathways, it modulates plant growth, development, and stress responses [15,16,17]. *HERK2* is a receptor kinase, regulated by brassinosteroid, which is essential for cell elongation during vegetative growth [18]. *SIF2* participates in *MAMP*-mediated stomatal immunity by interacting with the *BAK1/FLS2* complex, leading to its phosphorylation and subsequent activation of *SLAC1*. Notably, *SLAC1* expression is strongly induced during leaf senescence [19].

Rice (*Oryza sativa* L.) is a key global staple crop, accounting for the primary sustenance of nearly half the world’s population [20]. However, soil salinization has emerged as a primary abiotic stressor that severely limits rice productivity [21]. Osmotic stress, ion toxicity, and oxidative stress are among the primary mechanisms through which salt stress adversely affects rice growth and development. These disruptions result in reduced photosynthetic efficiency, stunted growth, decreased tillering, and ultimately, significant losses in both yield and quality [22,23,24]. Given this, a primary research objective is to identify key salt tolerance genes, which is crucial for advancing molecular breeding efforts directed toward creating stress-resistant rice varieties.

This study aims to conduct a genome-wide identification of the *OsCrRLK1L* gene family in rice and to investigate its fundamental functional attributes. Following the genome-wide identification of the *OsCrRLK1L* members, a systematic investigation of their evolutionary relationships, gene structures, and conserved motifs was performed, revealing the family’s evolutionary patterns. Furthermore, the expansion mechanisms and potential functions of this gene family were investigated by synthesizing evidence from gene duplication events, cis-acting element distributions, and GO/KEGG enrichment analyses. Key candidate genes governing salt stress responses were identified through expression profiling of the *OsCrRLK1L* family, thereby paving the way for mechanistic studies and supplying essential genetic resources for understanding abiotic stress adaptation in rice.

## 2. Materials and Methods

### 2.1. Genome-Wide Identification of the CrRLK1L Gene Family in Rice

For this study, the genomic data, including whole-genome sequences and annotation files for rice and *Arabidopsis thaliana*, were obtained from Ensembl Plants [25]. *CrRLK1L* genes in rice and *Arabidopsis* were identified with the aid of HMMER (version 3.3.2) and its corresponding Pfam models (PF12819 and PF07714), using an E-value cutoff of 1 × 10^−5^ [26]. All identified *CrRLK1L* protein sequences were further validated against the InterProScan [27], CDD [28], and SMART [29] databases. The isoelectric point, molecular weight, instability index, grand average of hydropathicity (GRAVY), and aliphatic index of OsCrRLK1Ls proteins were predicted using the Expasy ProtParam database [30].

### 2.2. Phylogenetic Analysis of the CrRLK1L Family

A phylogenetic tree was built to assess the evolutionary relationships of *CrRLK1L* genes across species; it was constructed via the neighbor-joining method based on a multiple sequence alignment of the protein sequences from Arabidopsis (61 sequences) and rice (36 sequences), performed with ClustalW [31] and was visualized using ggtree.

### 2.3. Analysis of Gene Structure, Conserved Motifs, and Phylogeny

The MEME suite was employed to predict the conserved motifs present in the 36 identified *OsCrRLK1L* proteins [32]. The determination of gene structures was achieved through the alignment of their genomic DNA and mRNA sequences. A phylogenetic tree was generated from the *OsCrRLK1L* protein sequences with the neighbor-joining (NJ) method in MEGA12 [33]. Both the gene structures and the phylogenetic tree were visualized using R, with the aid of the ggtree package.

### 2.4. Prediction of Cis-Regulatory Elements in Promoter Regions

For the prediction of cis-acting elements, the 2 kb genomic DNA sequences upstream of the start codon (ATG) in the promoters of *OsCrRLK1L* genes were extracted and submitted to the PlantCARE database (http://bioinformatics.psb.ugent.be/webtools/plantcare/html/ accessed on 15 October 2025) for analysis. The abundance of the identified elements was subsequently visualized with the R package ggplot2 (v.4.0.0).

### 2.5. Genome-Wide Duplication of the Rice CrRLK1L

The chromosomal locations and relative distances among *OsCrRLK1L* gene family members were determined based on information extracted from rice GFF files. WGD events were identified by MCScanX (v.1.0.0) [34]. WGD includes segmental duplications and tandem duplications. For a WGD event to be confirmed, it was required that the shorter sequence cover over 70% of the longer sequence and that the aligned regions exhibit more than 70% sequence similarity [35]. Two genes situated on the same chromosomal segment and separated by less than 100 kb are defined as a tandem duplication [36]. Segmental duplication refers to two genes that underwent polyploidization followed by chromosomal rearrangement [37]. Visualization was performed using Circos (v.0.69) [38]. The Ka values for each duplicated *OsCrRLK1L* gene pair were finally calculated using the KaKs_Calculator 2.0 program.

### 2.6. Gene Expression Pattern Analysis

This study leveraged publicly available RNA-seq datasets from the NCBI GEO to examine *OsCrRLK1L* gene family expression. The dataset under accession number GSE103300 [39] provided profiles across diverse rice tissues, and GSE206706 [40] was interrogated to elucidate expression under salt stress conditions. Functional annotation of the identified rice proteins was performed utilizing the eggNOG-mapper database [41]. To identify significantly enriched GO terms and KEGG pathways (*p* < 0.05), the annotation results were subjected to enrichment analysis using the clusterProfiler package (v.4.10.1) in R. The results were visualized using the same package.

### 2.7. Plant Growth Conditions and Treatments

The rice cultivar Zhonghua 11 (ZH11) was used to analyze the relative expression levels of *OsCrRLK1L* under salt stress. Mature, healthy seeds were surface-sterilized by immersion in 1% NaClO for 15 min, thoroughly rinsed with distilled water, and sown on agar medium. Seedlings were transplanted into Hoagland’s solution and cultivated under a 14 h light/10 h dark photoperiod, with corresponding temperatures of 25 °C and 23 °C. Once they reached the three-leaf stage, uniformly developed individuals were exposed to salt stress by applying 100 mM NaCl. Following treatment, leaf and root samples were collected at 0, 1, 3, 6, 12, and 24 h, immediately frozen in liquid nitrogen, and stored at −80 °C. Every treatment group consisted of no fewer than three biological replicates.

### 2.8. RT-qPCR Validation

Total RNA was isolated using the UltraPure Total RNA Extraction Kit (Hangzhou Sumgen Biotech Co., Ltd., Hangzhou, China) and stored at −80 °C. RNA purity and concentration were assessed on a NanoDrop spectrophotometer (Thermo Fisher Scientific, Waltham, MA, USA), and only samples with an A260/A280 ratio between 1.8 and 2.1 were processed for cDNA synthesis. First-strand cDNA was synthesized in a 10 μL reaction volume using PrimeScript™ RT Mix (Takara Biomedical Technology (Beijing) Co., Ltd., Beijing, China). as per the manufacturer’s protocol. Quantitative real-time PCR (RT-qPCR) was conducted on a LightCycler 96 system (Roche, Switzerland) using SYBR Green chemistry (Vazyme Biotech Co., Ltd., Nanjing, China). *OsActin* was used as the internal reference gene [14], and relative gene expression was calculated by the 2^–ΔΔCT^ method [42]. All reactions were run in triplicate, with primer sequences and amplicon lengths detailed in Table S1.

## 3. Results

### 3.1. Identification and Physicochemical Characterization of the CrRLK1L Gene Family

**Figure 1:**
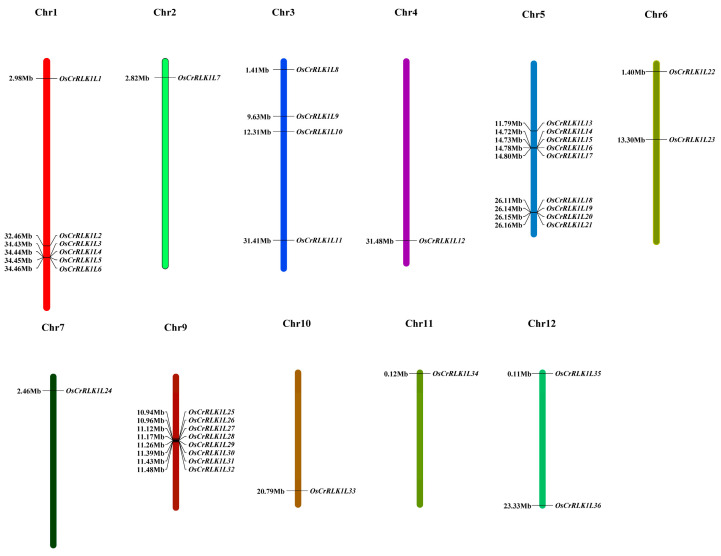
Distribution of the CrRLK1L gene on the rice chromosome.

The rice genome was found to harbor 36 CrRLK1L genes, which are unevenly distributed across its 11 chromosomes (Table S2). Based on their chromosomal locations, these genes were systematically designated as OsCrRLK1L1 to OsCrRLK1L36 (Figure 1). An analysis of the physicochemical properties was conducted for all 36 OsCrRLK1L family members (Table S2). The OsCrRLK1L proteins exhibited a broad molecular weight span from 60.15 kDa (OsCrRLK1L28) to 105.96 kDa (OsCrRLK1L36), while their theoretical pI values were mostly concentrated in the weakly acidic region of 5.5–6.5, indicative of substantial family diversity. However, a few members, such as OsCrRLK1L9 (8.71), are basic. This suggests potential localization in different cellular compartments. The instability index is below 40 for 68.8% of OsCrRLK1L proteins, indicating that the majority of them are relatively stable. Among these, OsCrRLK1L16 (30.35) exhibits the highest stability, while OsCrRLK1L4 (52.78) is the least stable. The aliphatic indices ranged from 73.23 to 98.01, with the majority falling between 80 and 90. This implies that most proteins in this family exhibit good thermal stability. OsCrRLK1L16 showed the highest value (98.01), corresponding to the greatest thermal stability. All OsCrRLK1L proteins displayed negative GRAVY values (ranging from −0.036 to −0.455), suggesting that they are hydrophilic. Among them, OsCrRLK1L19 was the most hydrophilic (−0.455). These physicochemical properties are thus central to elucidating the structure and function of the CrRLK1L protein family.

### 3.2. Phylogenetic Analysis of CrRLK1Ls

**Figure 2:**
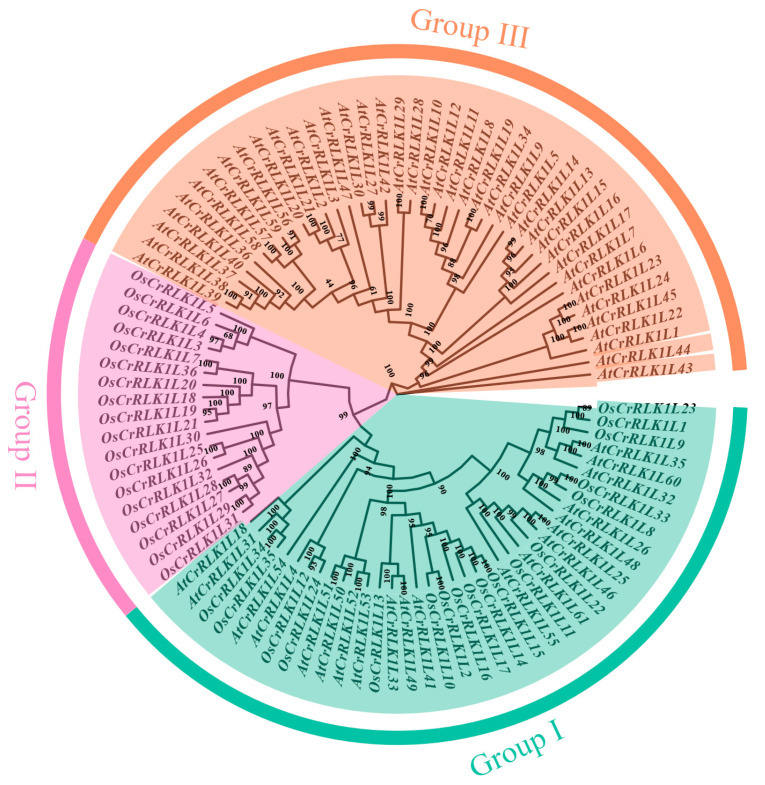
Phylogenetic tree of *CrRLK1Ls* in rice and Arabidopsis.

To infer the evolutionary relationships, a phylogenetic analysis was performed using the protein sequences of the 36 rice *OsCrRLK1Ls* and 61 from *Arabidopsis* (*AtCrRLK1Ls*) (Tables S2 and S3). All sequences are clustered into three subfamilies (Figure 2). Group II and Group III comprise 18 and 41 *CrRLK1Ls* proteins, respectively. All *CrRLK1Ls* in Group II are from rice, while all *CrRLK1Ls* in Group III are from *Arabidopsis*. Phylogenetic analysis revealed that 38 *CrRLK1L* genes from rice and *Arabidopsis* form Group I, implying a shared evolutionary history for these family members across the two species.

### 3.3. Protein Domain and Gene Structure Analysis of OsCrRLK1Ls

**Figure 3:**
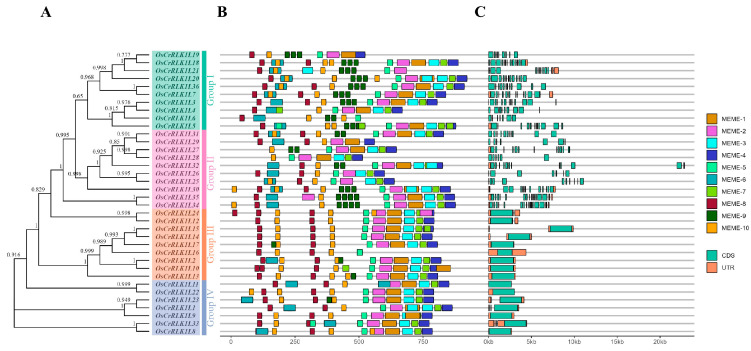
Gene structure and motif analysis of the rice *CrRLK1L* family. (**A**): Phylogenetic tree showing that *OsCrRLK1Ls* were classified into four distinct clades. (**B**): Distribution of conserved motifs in *OsCrRLK1L* proteins. Fifteen distinct motifs are represented by color-coded boxes. (**C**): Exon–intron structure of *OsCrRLK1L* genes. Green rectangles represent exons (CDS), orange rectangles indicate untranslated regions (UTRs), and thin gray lines connecting them represent introns.

The *OsCrRLK1L* gene information was extracted from the rice reference genome sequence and its annotation files to display its gene structure. Our results indicate that the evolutionary branches, Group I and Group II, share similar exon–intron compositions, while Group III and Group IV form a distinct cluster with comparable characteristics (Figure 3A,C). Each gene in Groups I and II contains at least three exons. In contrast, genes in Groups III and IV, which share a similar structural pattern, each contain only one exon. This structural composition indicates a high degree of conservation among genes within the same subgroup. We predicted conserved motifs in the *OsCrRLK1L* proteins and identified a total of 10 motifs (Figure 3A,B; Table S4). The number of motifs present in each protein ranged from 5 (*OsCrRLK1L16*) to 16 (*OsCrRLK1L5* and *OsCrRLK1L30*). Further analysis revealed significant similarity in motif composition among *OsCrRLK1L* proteins within the same subgroup, suggesting potential functional conservation among these members. Notably, the motif distribution was similar between Group I and Group II, as well as between Group III and Group IV, further supporting the consistency between structural gene clustering and motif composition.

### 3.4. Analysis of the OsCrRLK1L Gene Promoter

**Figure 4:**
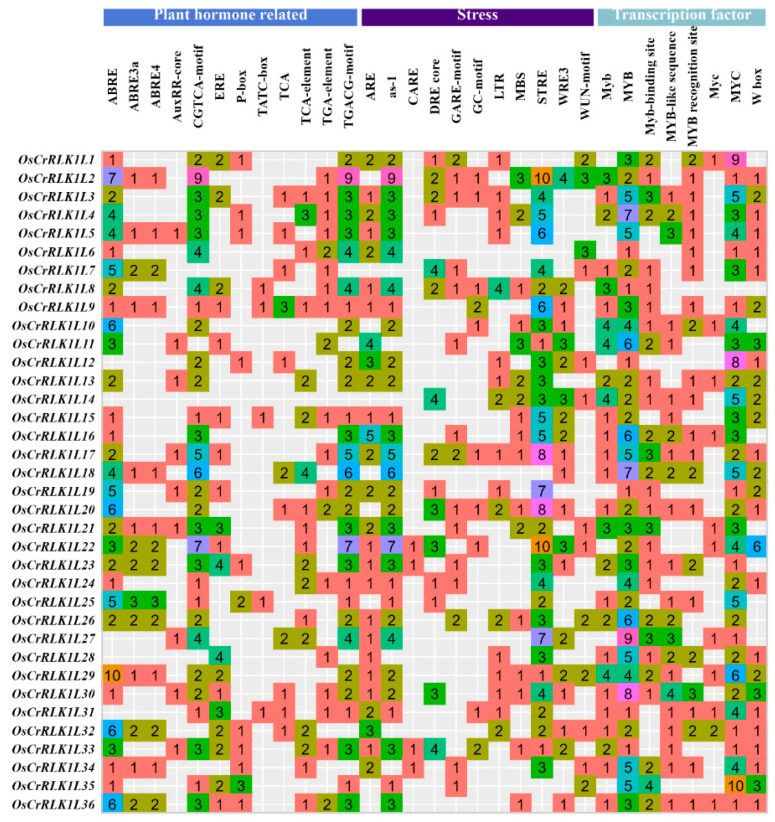
Promoter analysis of *OsCrRLK1Ls*. The color bar indicates the number of cis-acting elements identified in each sequence.

Analysis of the 2.0 kb promoter regions of the 36 *OsCrRLK1L* genes using PlantCARE revealed cis-regulatory elements related to three key functions: plant hormone signaling, abiotic stress responses, and transcription factor binding (Figure 4; Table S5). The promoter regions of the 36 *OsCrRLK1Ls* are enriched with various cis-acting elements associated with abiotic stress and hormone responses. Identified stress-related elements include anaerobic (ARE, as −1), low-temperature (LTR), stress tolerance (STRE), and drought (MBS)-response elements. Hormone-related elements encompass those responsive to abscisic acid (ABA), salicylic acid (SA), methyl jasmonate (MeJA), gibberellic acid (GA), ethylene (ERE), and auxin (IAA). Among them, the ABA (ABRE, ABRE2, ABRE3a, and ABRE4), SA (TCA, TCA-element, and TGA-element), and MeJA (CGTCA-motif and TGACG-motif) response elements are the most widely distributed and abundant. Transcription factor binding sites are predominantly associated with the MYB and MYC families, implicating a broad capacity of *OsCrRLK1Ls* to participate in diverse plant regulatory processes.

### 3.5. Analysis of Whole-Genome Duplication in the OsCrRLK1L Gene Family

**Figure 5:**
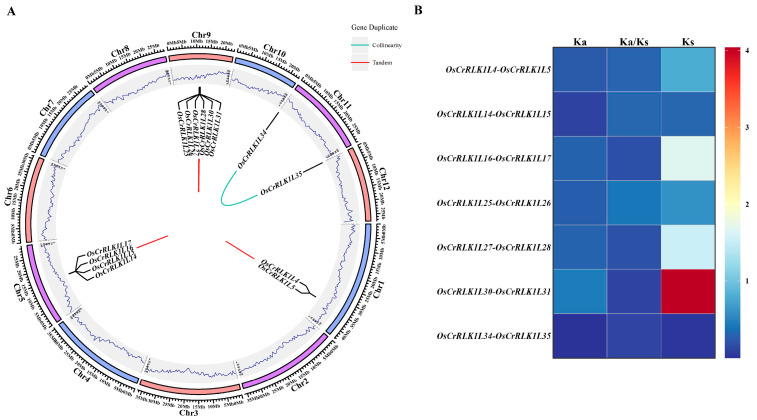
Duplication event analysis of *OsCrRLK1L* gene. (**A**): Analysis of duplication events in the *OsCrRLK1L* gene family. (**B**): Ka/Ks analysis of repetitive events between *OsCrRLK1Ls*. Red and blue lines indicate tandem and segmental duplications, respectively.

Whole-genome duplication (WGD) furnishes the genetic raw material for the morphological and physiological innovations that underpin plant evolution. To investigate this phenomenon at the gene family level, we analyzed the *OsCrRLK1L* genes and identified duplication events in 14 members across five chromosomes (Figure 5A). Among these, we identified 12 tandem repeat sequences (*OsCrRLK1L25/26/27/28/29/30*, *OsCrRLK1L14/15/16/17* and *OsCrRLK1L4/5*), while two genes (*OsCrRLK1L34/35*) underwent segmental duplication. Our findings suggest that tandem duplication has been a major force behind the expansion of the *OsCrRLK1L* gene family. We subsequently estimated the selective pressure on seven duplicated gene pairs by determining their Ka and Ks substitution rates. As shown in the figure, the Ka/Ks ratios for all detected gene pairs (e.g., *OsCrRLK1L14-OsCrRLK1L15* and *OsCrRLK1L16-OsCrRLK1L17*) were significantly less than one, ranging from 0.11 to 0.41 (Figure 5B; Table S6). This result indicates that these genes underwent intense purifying selection following duplication.

### 3.6. Analysis of Expression Patterns of OsCrRLK1Ls in Different Tissues

**Figure 6:**
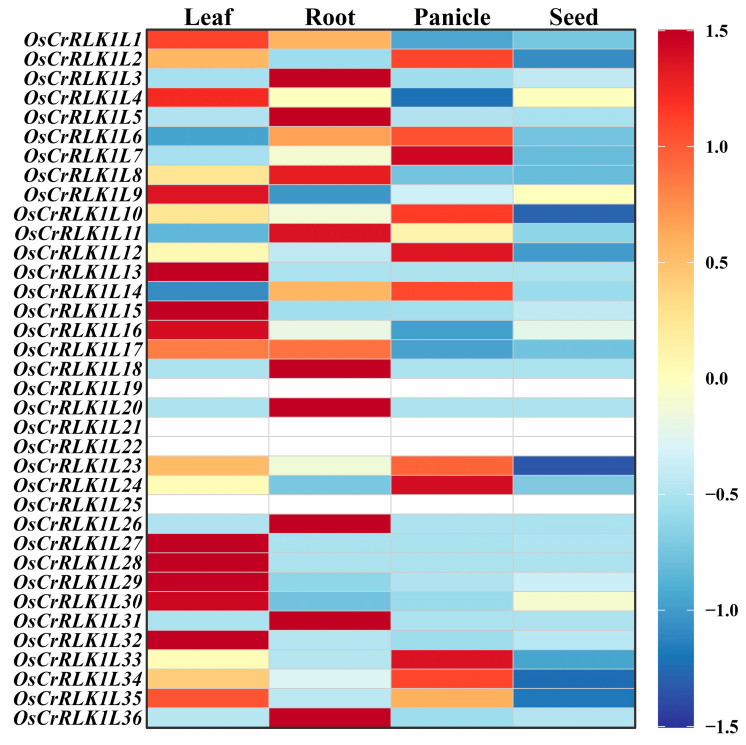
Tissue-specific expression patterns of OsCrRLK1L genes in rice. The color gradient from red to blue corresponds to high and low transcript abundance, respectively.

To elucidate the tissue-specific regulatory patterns of the *OsCrRLK1L* gene family, whose members often have distinct roles in plant development, we analyzed their transcriptional abundance in leaves, roots, panicles, and seeds. We successfully measured the expression of 32 out of the 36 genes (Figure 6). Results indicate that the *OsCrRLK1L* genes exhibit significant tissue specificity across various rice organs. Twelve members show high expression in leaves, nine in roots, and nine in panicles, whereas the lowest expression levels are consistently observed in seeds (Figure 6; Table S7).

### 3.7. Expression Pattern and Enrichment Analysis of the OsCrRLK1L Gene Family in Response to Salt Stress

**Figure 7:**
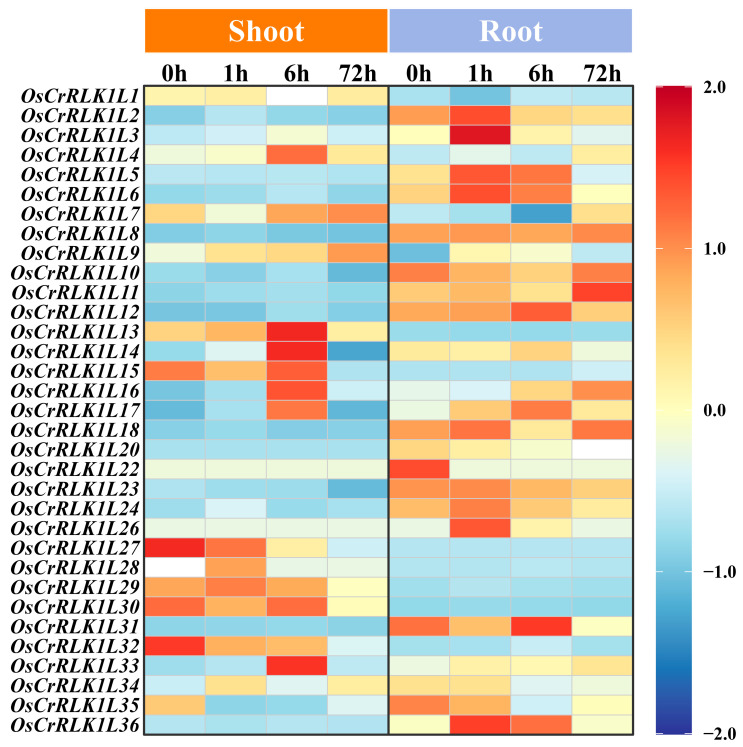
Temporal expression patterns of *OsCrRLK1L* genes under salt stress. The color scale from red to blue represents high to low expression levels.

To elucidate the function of *OsCrRLK1L* under salt stress, we performed a time-course analysis of its expression dynamics under 100 mM NaCl treatment. Analysis revealed the transcriptional abundance of 36 *OsCrRLK1L* genes (Figure 7; Table S8). Results indicate that under continuous salt stress, *OsCrRLK1L* genes exhibited distinct expression patterns. In shoots, eight genes (*OsCrRLK1L4/13/14/15/16/17/30/33*) reached their peak expression levels at 1 h. In contrast, a different set of eight genes (*OsCrRLK1L2/3/5/6/23/24/26/36*) peaked in roots at the same time-point. Notably, this latter group showed only minimal induction in shoots under salt stress.

To gain functional insights into the *OsCrRLK1L* family, a comprehensive GO and KEGG pathway enrichment analysis (*p* < 0.05) was performed for all its members. Results show significant enrichment of *OsCrRLK1L2*, *OsCrRLK1L10*, *OsCrRLK1L14*, *OsCrRLK1L15*, *OsCrRLK1L16*, and *OsCrRLK1L17* in established salt-tolerance mechanisms, encompassing hormone signaling pathways for brassinolide and abscisic acid, root morphogenesis, stomatal regulation, and cell wall modification (Figure S1; Table S9). KEGG enrichment analysis implicates *OsCrRLK1L3* and *OsCrRLK1L5* in the MAPK signaling pathway (Figure S1). These findings point to critical and complex functions for genes within this family in underlying rice salt stress tolerance.

**Figure 8:**
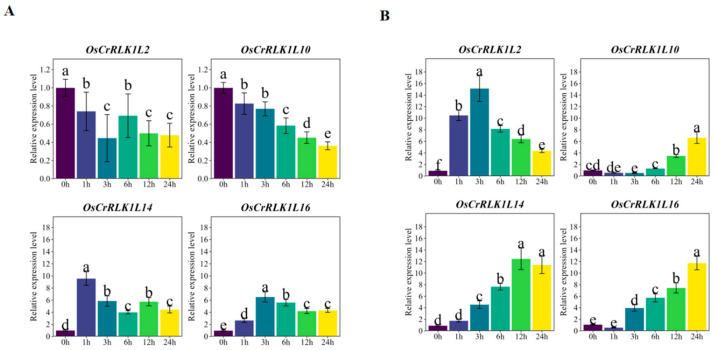
RT-qPCR analysis of OsCrRLK1L gene expression patterns under salt stress conditions. (**A**): Expression levels of OsCrRLK1Ls in the shoot under salt stress within 24 h; (**B**): expression levels of OsCrRLK1Ls in the root under salt stress within 24 h. Values are the mean ± SE of three replicates, and statistically significant differences (*p* < 0.05) among groups, as determined by LSD test, are indicated by different lowercase letters (a–e), with bars sharing the same letter being not significantly different.

To investigate the salt stress response of *OsCrRLK1L* genes, we selected four members (*OsCrRLK1L2/10/14/16*) for RT-qPCR analysis, guided by their expression patterns and enrichment results. ZH11 seedlings were subjected to 100 mM NaCl stress, following which RNA was isolated from their leaf and root tissues at 0, 1, 3, 6, 12, and 24 h after the treatment. The results indicate that under salt stress, *OsCrRLK1Ls* exhibit varying degrees of induction. Notably, *OsCrRLK1L14* and *OsCrRLK1L16* were induced in both shoots and roots (Figure 8A), whereas *OsCrRLK1L2* and *OsCrRLK1L10* displayed divergent expression patterns between these tissues (Figure 8B). This spatial specificity suggests distinct biological roles for these genes in salt stress adaptation.

## 4. Discussion

The emergence of reference genomes for numerous plant species has enabled the identification of many gene families [43,44,45,46]; however, a comprehensive characterization of the *CrRLK1L* gene family in rice is still lacking. This study encompassed a comprehensive analysis of the *OsCrRLK1L* gene family, focusing on their phylogenetic relationships, chromosomal distributions, conserved motifs, gene structures, WGD events, putative promoter CREs, expression profiles, and responses to salt stress. Members of the *CrRLK1L* gene family are characterized by the presence of both a conserved melittin-like domain and a conserved kinase domain [47]. Using the characteristic domains of the *CrRLK1L* gene family, this study identified 61 and 36 members in the Arabidopsis and rice genomes, respectively. Approximately 150 million years ago, monocotyledons and eudicots diverged from a common ancestor, embarking on separate evolutionary paths [48]. Subsequently, eudicots underwent a major expansion around 100 million years ago, likely driven by a polyploidy event [49]. To gain insights into the evolutionary trajectory of the *CrRLK1L* gene family, a phylogenetic tree was reconstructed for rice and Arabidopsis, accompanied by a clustering analysis (Figure 2). Phylogenetic analysis revealed that Group II comprises exclusively rice *CrRLK1L* genes, whereas Group III contains only those from *Arabidopsis* (Figure 2). This distribution pattern suggests that subsequent to the divergence of monocots (rice) and dicots (*Arabidopsis*), the *CrRLK1L* gene family underwent independent, lineage-specific expansion in each species. These duplication events led to the retention of paralogous genes, which may have acquired novel functions via distinct evolutionary paths. The presence of Group I subclusters containing *CrRLK1L* genes from both rice and Arabidopsis indicates that these genes represent the phylogenetically ancient and functionally conserved core of the *CrRLK1L* family. Originating from the last common ancestor of these two species, they have been retained throughout their independent evolutionary paths. This conservation suggests that they perform essential, core functions such as regulating fundamental cellular signaling and growth processes, which are fundamental to this kinase family. Previous studies indicate that genes within the same subgroup tend to share similar functional roles [50]. In Group I, *AtCrRLK1L4* (*AT1G30570*) is crucial for cell elongation during vegetative growth [51]. *AtCrRLK1L22* (*AT2G19190*) is involved in early defense signaling and is strongly induced during leaf senescence [52]. *AtCrRLK1L31*(*AT2G37050*) regulates salt-induced proteasome maturation through *UMP1A* phosphorylation and plays a key role in salt stress responses [53]. Meanwhile, *AtCrRLK1L51* (*AT5G39000*) acts as a negative regulator of apical growth in nuclear cells; mutants exhibit defects in pollen germination, pollen tube elongation, and root hair development [54]. These findings collectively imply that *OsCrRLK1L* members are also integral to diverse abiotic stress responses.

The exon–intron structure of a gene significantly influences its function by affecting RNA splicing, stability, and chromatin organization [55,56,57]. From an evolutionary perspective, gene families often accumulate more introns during their early stages of expansion. Subsequently, in response to environmental pressures, their structures may become more compact through intron loss. This structural simplification is thought to facilitate rapid gene activation, allowing for timely responses to environmental stressors [58,59,60]. Among all 36 identified *OsCrRLK1Ls*, 16 possess a single exon and 20 contain multiple exons (Figure 3). Notably, the single-exon genes were rapidly and strongly induced under salt stress (Figure 7), suggesting that structural simplicity may contribute to efficient stress responses.

Gene duplication is a fundamental driving force in genome and species evolution. It provides the raw material for evolutionary innovation, and these duplicated genes can ultimately contribute to biological diversity through mechanisms such as neofunctionalization [61]. During evolution, an ancestral single-copy gene expanded into a multigene family following a whole-genome duplication event [62]. Tandem duplication and segmental duplication are closely associated with the amplification of stress-related genes [63]. This process represents a stress response mechanism that enables plants to counteract environmental and biological damage [64]. Our analysis identified 12 tandem and 2 segmental duplications among the 36 *OsCrRLK1Ls*, indicating that tandem duplication is the primary driver for this family’s expansion. This mechanism typically leads to a clustered genomic distribution. The resultant genetic redundancy provides the evolutionary substrate for functional innovation, where gene copies can acquire novel or specialized roles through subfunctionalization [65]. Further Ka/Ks analysis showed that all eight WGD gene pairs had Ka/Ks ratios significantly less than one (Figure 5 A,B). This indicates that the protein sequences encoded by these eight WGD gene pairs are highly constrained, with most amino acid changes being deleterious and thus eliminated by purifying selection. Consequently, these genes likely perform essential, non-redundant biological functions within the *OsCrRLK1L* family, whose integrity is so critical for rice survival that it has led to their strict sequence conservation over evolution.

Cis-acting elements are key regulatory sequences that mediate hormone signaling to gene expression [66]. Among them, ABRE, ABRE3a, and ABRE4 are core ABA response elements; the presence of multiple ABREs or their combination with a coupling element enhances the expression of ABA-responsive genes, thereby improving plant stress tolerance [67,68,69]. Furthermore, binding sites for transcription factors like *MYC* and *MYB* are critical, as they can synergize with ABREs to co-regulate ABA signaling in stress-inducible genes such as *RD22* [70]. SA plays a crucial role in regulating plant growth and defense. One key mechanism is the induction of defense genes, which depends on the cooperative interaction between specific cis-acting elements and transcription factors [71]. A well-established example involves the *TGA* transcription factor, which specifically binds to the TGA element to activate SA-responsive genes, thereby enhancing plant tolerance to abiotic stress [72]. Similarly, the response to MeJA relies on the interaction between its specific cis-acting elements and transcription factors such as *MYB*, thereby initiating the expression of downstream defense genes [73,74]. In this study, we identified abundant binding sites for ABA, SA, MeJA, *MYB*, and *MYC* in the 2 kb promoter regions of *OsCrRLK1Ls* (Figure 4). This suggests that the expression of *OsCrRLK1Ls* is likely precisely modulated by these hormone signals, with the output potentially being amplified and specified by key transcription factors like *MYB* and *MYC*, enabling this gene family to play a synergistic and crucial role in plant stress responses.

RT-qPCR validation under salt stress confirmed the transcriptomic profiles of key *OsCrRLK1L* members, which were induced yet tissue-specific. *OsCrRLK1L2* and *OsCrRLK1L10* showed a notable reciprocal regulation—upregulated in roots but downregulated in shoots—implying a mechanism for coordinating root–shoot dynamics to enhance whole-plant salt tolerance. The promoters of these genes are enriched with diverse hormone response elements, providing a regulatory foundation for this complex coordination. Thus, the *OsCrRLK1L* family is positioned as a key integrator of multiple hormonal signals to implement holistic salt stress adaptation in rice. While this study provides a comprehensive characterization of the *OsCrRLK1L* family, it is important to note that the functional implications discussed herein are largely inferential and await direct experimental confirmation in future studies.

## 5. Conclusions

Our systematic analysis identified 36 *CrRLK1L* genes in rice. We further demonstrate that this gene family exhibits high lineage-specific divergence between monocots and dicots, and that its expansion in rice was primarily driven by tandem duplication events. Further analysis identified three distinct types of cis-acting elements within the *OsCrRLK1L* promoters, supporting the gene’s role in abiotic stress responses and diverse regulatory processes in rice. Furthermore, exon numbers diverge substantially among *OsCrRLK1L* subgroups. Integrative data from RNA-seq, enrichment analysis, and RT-qPCR implicate OsCrRLK1L2, 10, 14, and 16 in salt stress responses. Collectively, our findings establish a foundation for the comprehensive functional exploration of the *OsCrRLK1L* gene family.

## Data Availability

The RNA-seq data for this study can be found in the National Center for Biotechnology Information under the accession numbers PRJNA401663 and PRJNA851797 (https://www.ncbi.nlm.nih.gov/ accessed on 20 October 2025).

## Supplementary Materials

The following supporting information is available online at https://www.mdpi.com/article/10.3390/genes16121454/s1, Figure S1: GO and KEGG enrichment analysis of *OsCrRLK1Ls*. Table S1: Primer sequences used for quantitative real-time PCR. Table S2: Physicochemical properties of the OsCrRLK1L proteins. Table S3: Gene list of 61 *CrRLK1L* homologs in *Arabidopsis thaliana*. Table S4: Identified motif sequences in *OsCrRLK1Ls*. Table S5: Cis-elements analysis of *OsCrRLK1L1* genes. Table S6: Ka/Ks analysis of duplicated *OsCrRLK1L* gene pairs. Table S7: Expression profiles of *OsCrRLK1Ls* in leaves, roots, spikes, and seeds. Table S8: Expression changes in the rice *CrRLK1Ls* in response to salt stress. Table S9: Rice *CrRLK1Ls* GO and KEGG enrichment.
