# Supplementary figures and images for: Identification and Functional Characterization of the CrRLK1L Gene Family in Salt Tolerance in Rice (Oryza sativa L.)
Source: Genes (Basel). 2025 Dec 4;16(12):1454. doi: 10.3390/genes16121454 (PMC12733022; doi:10.3390/genes16121454)

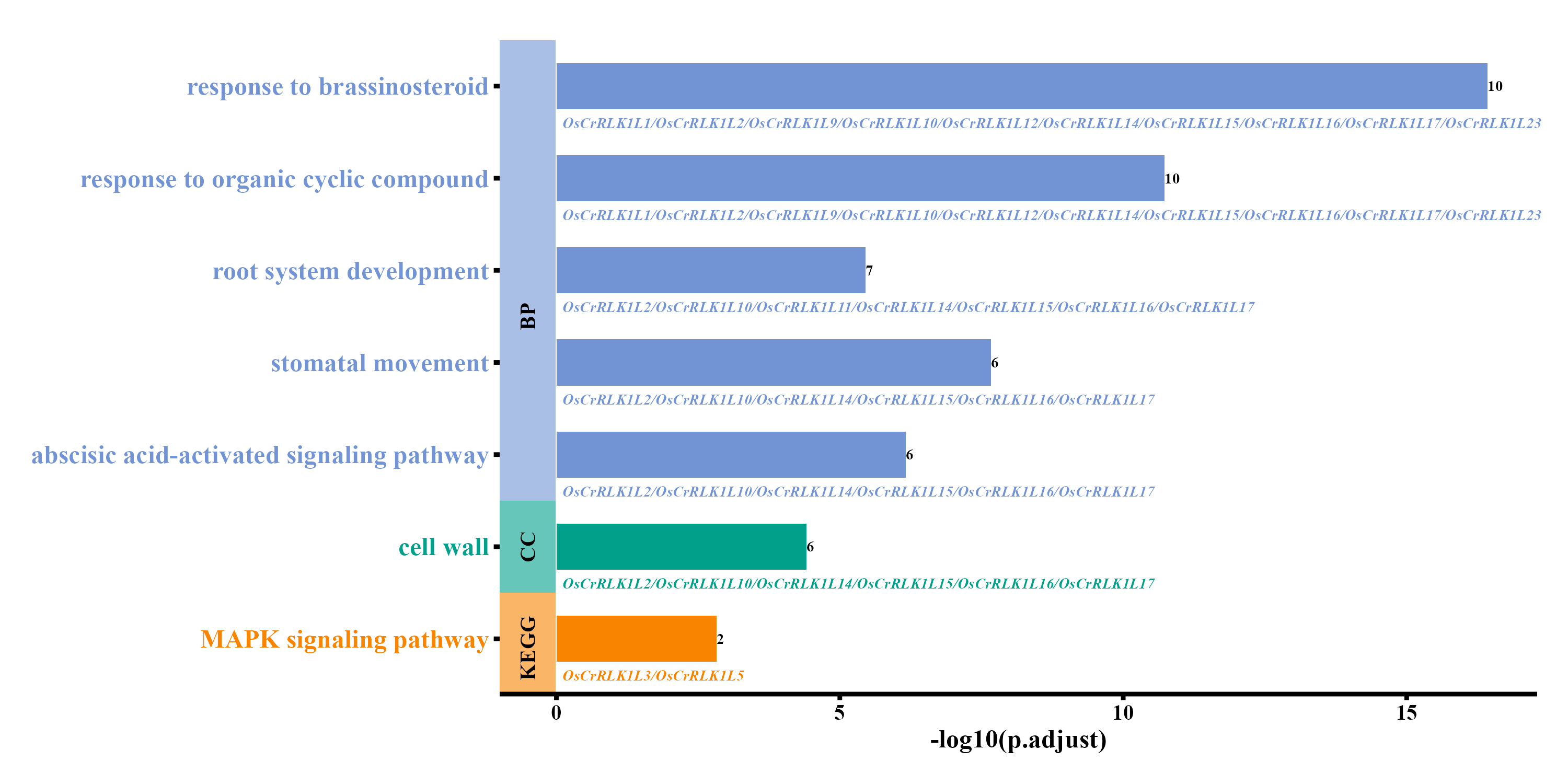

Supplement: Supplementary file 1 [file genes-16-01454-s001.zip › Supplementary Data/Figure S1.tif]
